# When Meningioma Travels: Rare Extracranial Metastasis—Case and Review

**DOI:** 10.1002/ccr3.72494

**Published:** 2026-04-10

**Authors:** Tala Najdi, Sami Abi Farraj, Pierre El Sett, Clarisse Kattan, Antoine El Sett, Antoine Chartouni, Patrick Ghorayeb, Ghadi Dagher, Rhea Khazen, Fares Azoury, Joseph Kattan, Hampig Kourie

**Affiliations:** ^1^ Department of Hematology‐Oncology Hotel‐Dieu de France University Hospital Beirut Lebanon; ^2^ Faculty of Medicine Saint Joseph University of Beirut Beirut Lebanon; ^3^ Department of Radiation Oncology Hotel‐Dieu de France University Hospital Beirut Lebanon; ^4^ Department of Pathology Hotel‐Dieu de France University Hospital Beirut Lebanon

**Keywords:** atypical meningioma, extracranial spread, hepatic metastasis, histologic transformation

## Abstract

Meningiomas are the most common primary intracranial tumors; extracranial metastases occur in < 1% of cases, most often to the lungs or bone. Hepatic spread is exceptionally rare and is more common in atypical or recurrent disease. A 73‐year‐old man with a recurrent left frontal atypical meningioma (Grade II, 2016 WHO CNS classification) developed a 4 × 3 cm hepatic lesion 12 years after initial diagnosis. Liver biopsy revealed spindle‐cell proliferation positive for EMA and negative for S100 protein, consistent with metastatic meningioma. Subsequent intracranial resection showed focal anaplastic transformation (Grade 3, 2021 WHO CNS classification) with Ki‐67 up to 20%. Treatment with Sandostatin and Everolimus stabilized the hepatic lesion, although intracranial recurrences persisted and the patient died during a surgical brain resection of a recurrence from a massive intraoperative hemorrhage. This exceptionally rare hepatic metastasis from an atypical meningioma emphasizes the possible need for long‐term systemic surveillance. Repeated recurrences, high mitotic activity, and histologic transformation may predict metastatic potential. Our review of the last decade identified only a few similar extracranial cases, supporting targeted imaging and individualized therapy in high‐risk meningiomas.

## Key Clinical Message

Extracranial metastasis of atypical meningioma is exceptionally rare. Recurrent tumors with rising mitotic activity or histologic progression may warrant systemic surveillance. Early detection of distant spread can guide timely intervention and individualized management in high‐risk patients, and selected patients may benefit from systemic therapies, although effects remain variable.

## Graphical abstract

Recurrent atypical meningioma with anaplastic transformation (Grade 3) developed delayed hepatic metastasis 12 years after diagnosis. Biopsy confirmed metastatic meningioma. Treatment with Sandostatin and Everolimus stabilized disease before progression, highlighting the need for long‐term surveillance in high‐risk cases.

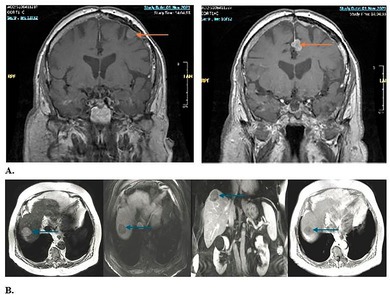

**Abbreviations**
ALT: alanine aminotransferase
AST: aspartate aminotransferase
CNS: central nervous system
EANO: European Association of Neuro‐Oncology
EMA: epithelial membrane antigen
F: female
Ga‐DOTATOC: gallium‐68 DOTA‐Tyr3‐octreotide
Gamma‐GT: gamma‐glutamyl transferase
H&E: hematoxylin and eosin
ICP: intracranial pressure
Ki‐67: proliferation index marker
M: male
MRI: magnetic resonance imaging
mTOR: mechanistic target of rapamycin
NM: not mentioned
PET/CT: positron emission tomography/computed tomography
S100: S100 protein
SRT: stereotactic radiotherapy
SSTR2: somatostatin receptor subtype 2
VMAT: volumetric modulated arc therapy
WHO: World Health Organization

## 1 Introduction

Meningiomas are the most common primary intracranial tumors, arising from arachnoid cap cells and accounting for approximately 20% of all intracranial neoplasms [1]. Most meningiomas are benign, and extracranial metastasis is exceedingly rare, with reported rates of less than 1% [2]. When metastases do occur, the lungs, bones, mediastinum, and lymph nodes are the predominant sites, while hepatic involvement is extremely uncommon, with only a few cases documented to date [3].

The risk of extracranial spread increases with higher histologic grade, elevated mitotic index, nuclear atypia, high cellularity, presence of necrosis, and repeated surgical interventions [4]. Male gender and tumor size have also been associated with metastatic potential [5]. Despite these risk factors, extracranial metastases remain exceptional, highlighting the need for vigilance in patients with atypical or recurrent meningiomas.

Here, we report the case of a 73‐year‐old man presenting with an asymptomatic solitary liver metastasis from a recurrent intracranial meningioma, 12 years after initial diagnosis. We also review recent literature on extracranial metastases of meningiomas over the past decade to contextualize this rare phenomenon and discuss implications for diagnosis, surveillance, and management.

## 2 Case Report

### 2.1 Case History

**FIGURE 1:**
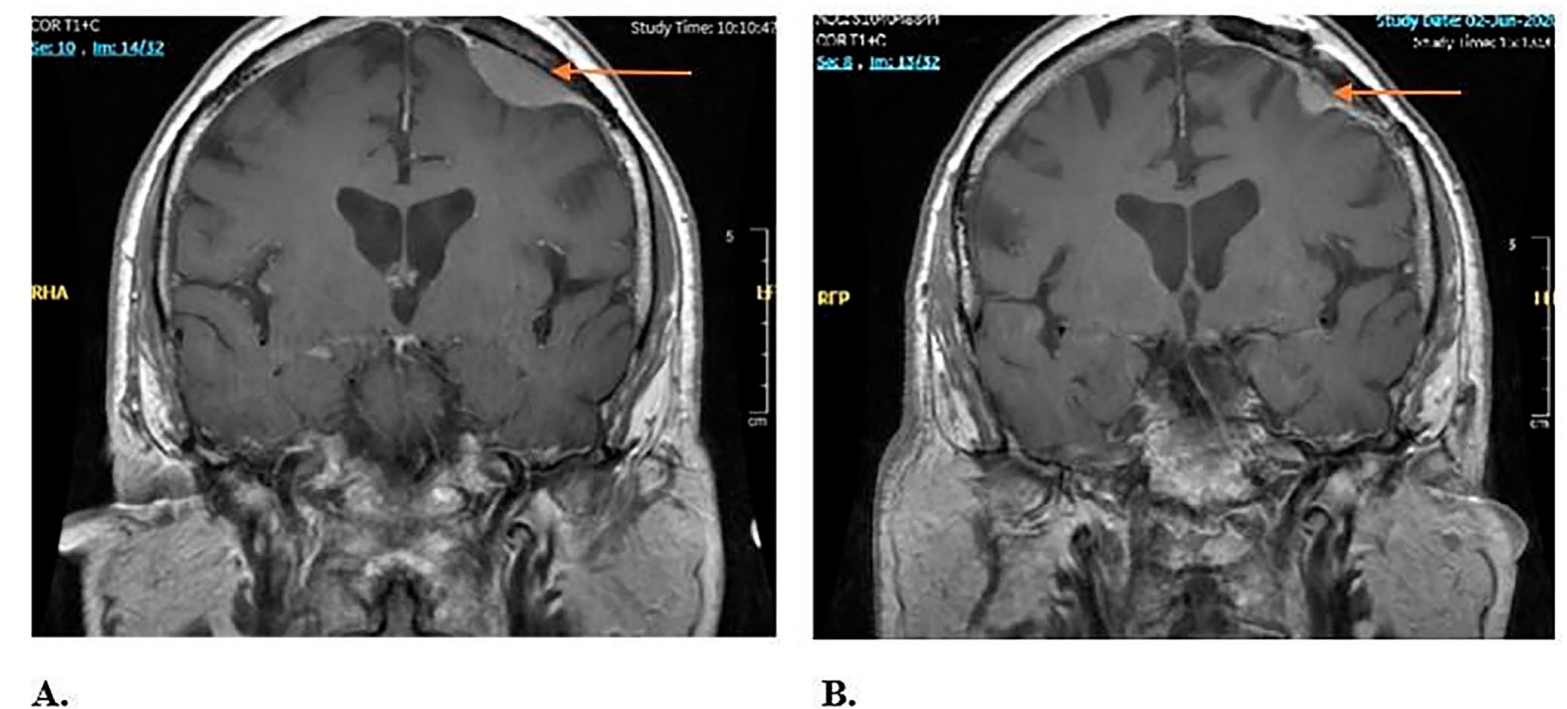
Brain MRI showing a left frontal meningioma. A: Initial MRI (September 2017) demonstrating a supero‐anterior left frontal meningioma. B: Follow‐up MRI (June 2020) showing a recurrent lesion at the same site.

A 73‐year‐old man with a history of hypertension, diabetes mellitus, and smoking presented to the emergency department in February 2022 with sudden‐onset dysarthria and Broca's aphasia, which began two hours before arrival. His neurological history was significant for a left frontal meningioma first diagnosed in August 2010, for which he underwent surgical resection; histopathology at that time revealed a transitional meningioma. Over the years, he remained under regular neurosurgical follow‐up, with serial imaging. The first recurrence occurred in September 2017, when he underwent a second resection for a recurrent left frontal tumor. Brain magnetic resonance imaging (MRI) performed at that time demonstrated a supero‐anterior left frontal meningioma (Figure 1A). Histopathological analysis identified the lesion as a meningothelial meningioma infiltrating the adjacent bone without cytologic anaplasia, showing a proliferation index marker (Ki‐67) averaging 10% with scattered hotspots of 25%–30% with world health organization (WHO) 2016 criteria for Grade I meningioma. The mitotic activity at that time did not meet the criteria for higher grades. In July 2019, he underwent a third resection, which was also confirmed as WHO grade I. In February 2020, a fourth surgery was performed for another local recurrence; histopathology revealing an atypical transitional meningioma with early signs of progression to WHO Grade II (2016 classification) and a heterogeneous Ki‐67 reaching 10%. During this period, the patient showed gradual improvement in neurological symptoms after each surgical resection, although he never fully returned to his baseline neurological function. Tumor recurrences were identified either on routine follow‐up imaging or following the re‐emergence of aphasia.

On follow‐up brain MRI in June 2020 (Figure 1.B), a small dural‐based lesion measuring 10 mm with homogeneous enhancement was observed, along with a more diffuse area causing thickening of the left parasagittal frontal dura, both consistent with small meningiomas. At that time, the patient was treated with volumetric modulated arc therapy (VMAT), receiving a total dose of 54 Gy delivered in 28 fractions. VMAT was well tolerated, with no acute neurological or systemic adverse effects reported, and the patient remained clinically stable for approximately one year following therapy. In addition, he underwent four cycles of Bevacizumab, completed in September 2021 to target tumor‐associated angiogenesis and help control edema.

**FIGURE 2:**
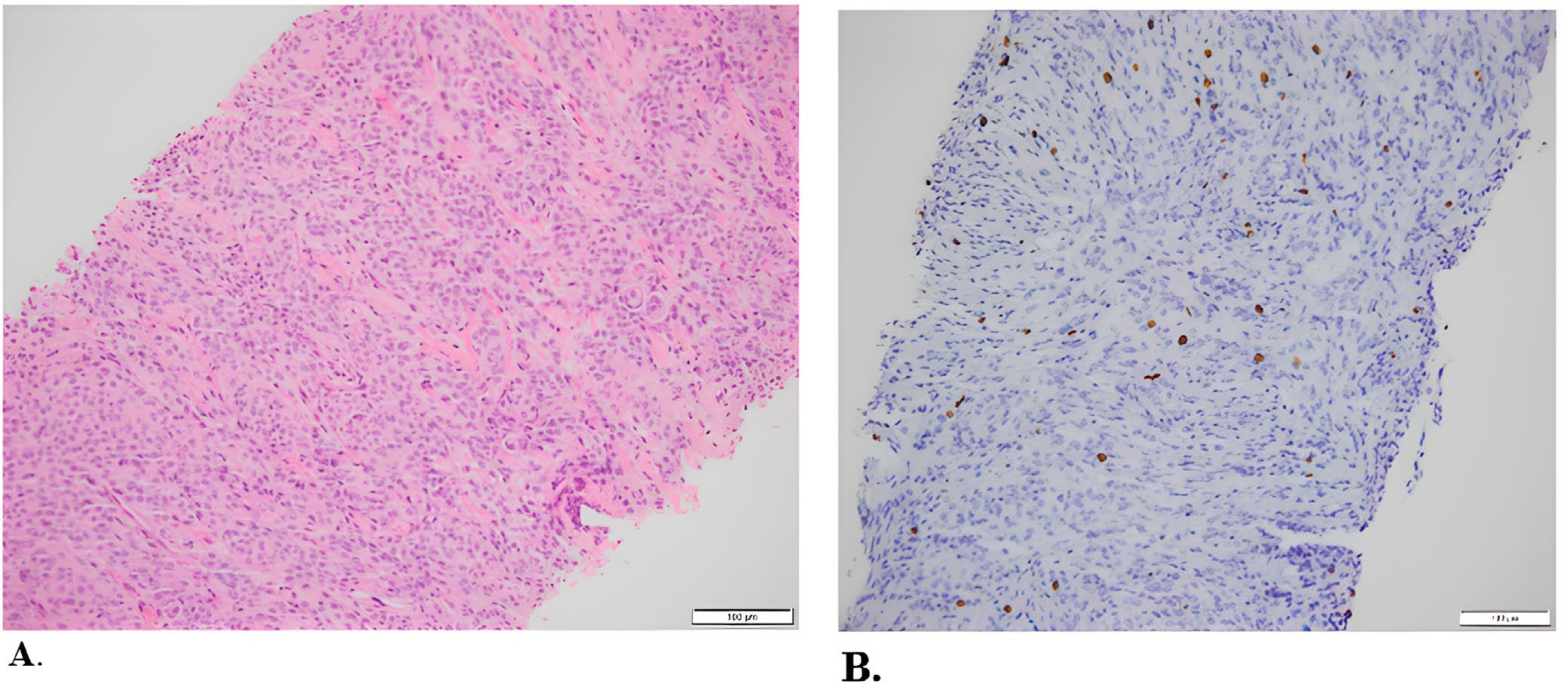
2017: Meningioma, grade 1. Core biopsies reveal whirling sheets of bland‐looking cells (A. H&E 20×). Ki67 is approximately 5%. (B. 20×).

**FIGURE 3:**
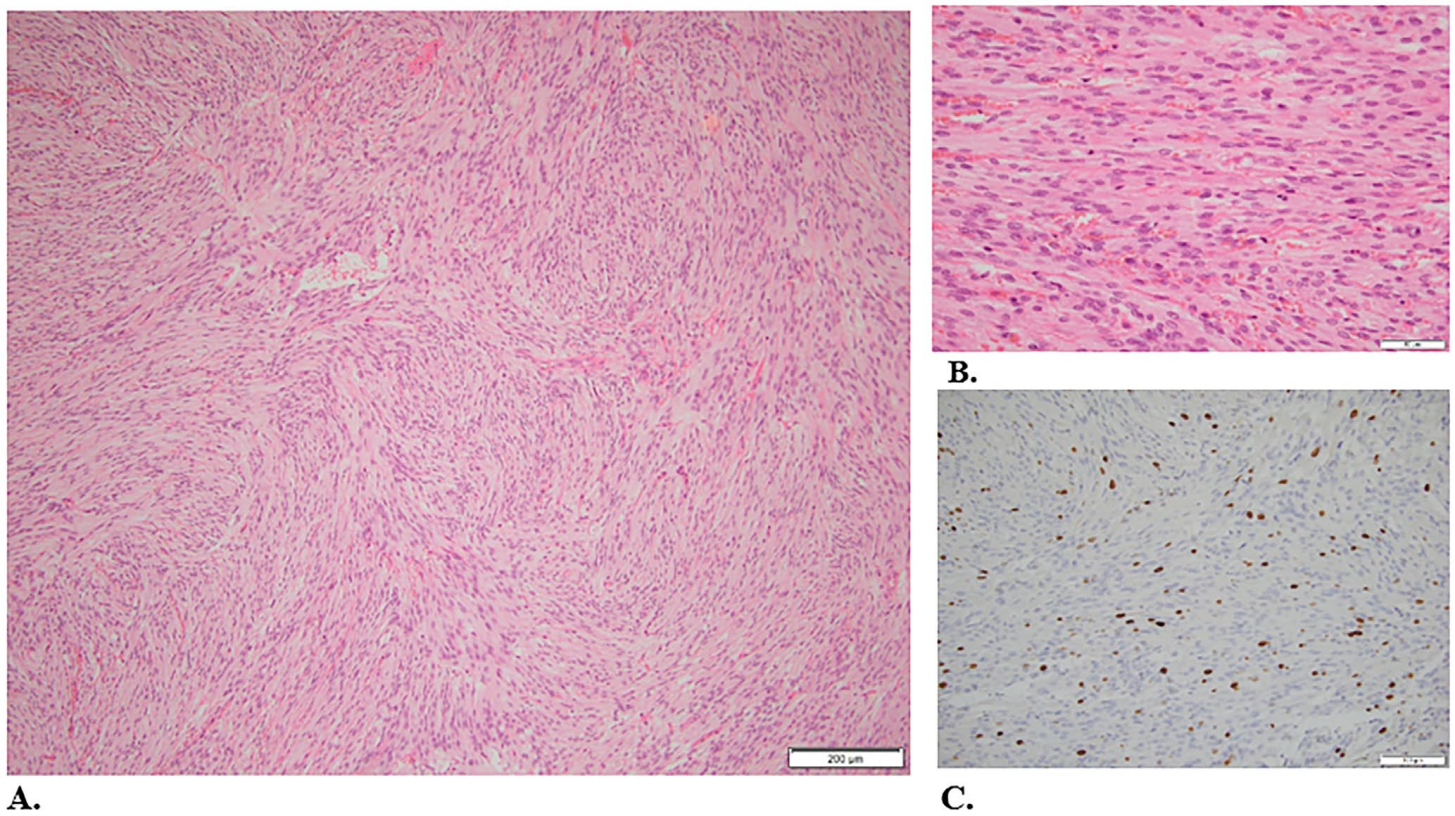
2020: Atypical meningioma, grade 2. The tumor shows a lobulated architecture with menigothelial whorls (A. H&E 10×). Despite cells showing indistinct cell membranes and round uniform nuclei, mitotic figures are increased (B. H&E 40×). Ki67 is approximately 10% (C. 20×).

Figures 2 and 3 show the histopathological features of the meningiomas resected in 2017 and 2020, respectively, stained with hematoxylin and eosin (H&E) and Ki‐67.

**FIGURE 4:**
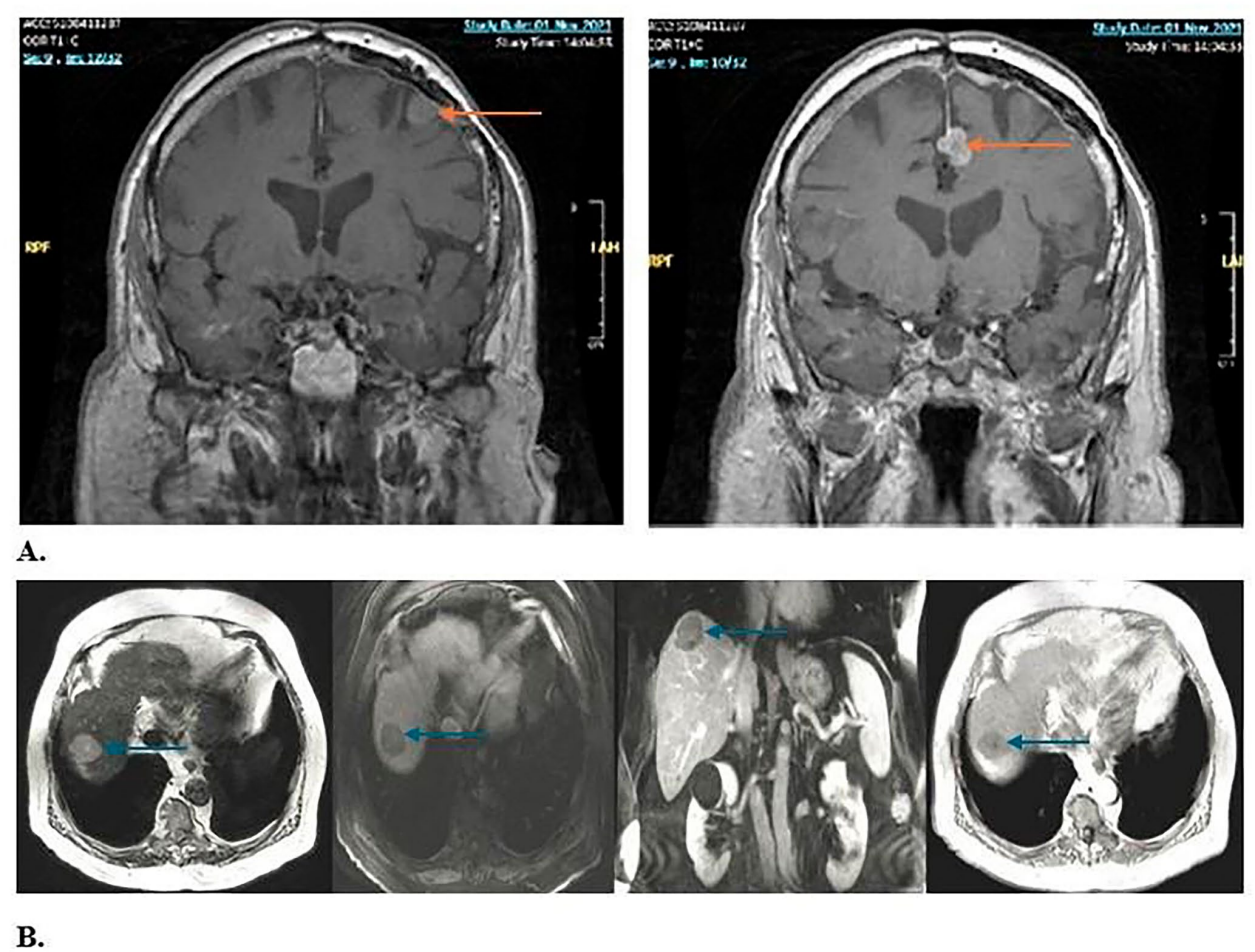
A. Brain MRI showing multiple meningiomatous lesions, November 2021. B. Abdominal MRI showing the hepatic metastasis of the meningioma.

However, in November 2021, the patient presented to the emergency department fully conscious, cooperative, and oriented, with a normal neurological and physical examination except for complete aphasia. Follow‐up MRI revealed progressive enlargement of meningiomatous lesions, despite previous radiotherapy (Figure 4.A). A left supero‐anterior frontal meningioma had increased to 13 mm. There was slight enlargement of the thickening of the left anterior para‐sagittal frontal dura, measuring 22 × 12 mm, and the left parafalcine region, measuring 8 mm. The left anterior paramedian frontal meningiomatous lesion adjacent to the superior frontal gyrus had enlarged to 15 mm, while a more extensive left frontal lesion involving the middle and inferior frontal gyri measured 35 × 27 × 37 mm. The patient was admitted for close monitoring and initiation of intravenous corticosteroid therapy. He subsequently underwent VMAT targeting the left inferior frontal lesion, receiving a total dose of 54 Gy delivered in 30 fractions, followed by additional bevacizumab therapy in this context of recurrence, which was continued until January 2022.

### 2.2 Laboratory and Imaging New Findings

In November 2022, the patient presented a different clinical picture, this time with fever. Laboratory evaluation revealed newly abnormal liver function tests, with ALT (alanine aminotransferase) 221 U/L, AST (aspartate aminotransferase) 123 U/L, Gamma‐GT (gamma‐glutamyl transferase) 399 U/L, and alkaline phosphatase 73 U/L, compared with normal results four months prior. Abdominal MRI demonstrated a dysmorphic liver with a 4 × 3.1 cm lesion on the hepatic dome, suspicious for malignancy (Figure 4.B). Percutaneous biopsy of the lesion revealed a tumoral proliferation of spindle cells with rare mitoses and mild atypia. Ki‐67 averaged 4% but reached 7% in some areas.

### 2.3 Diagnosis and Management

**FIGURE 5:**
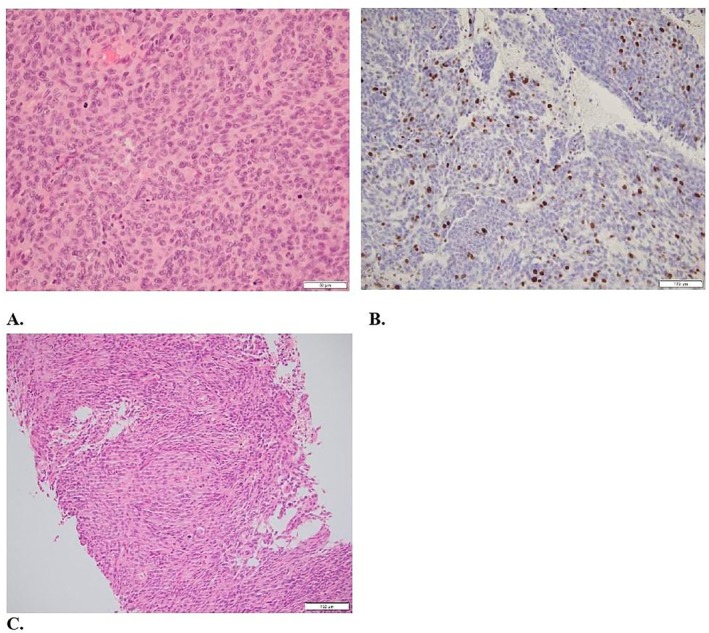
2022: Atypical meningioma, grade 2, with areas of anaplastic transformation (grade 3). The nuclear‐cytoplasmic ratio is increased, with prominent nuclei and increased mitotic figures (A. H&E 40×). Ki67 is up to 15%–20% in hotspots (B. 20×). (C) Liver biopsy reveals a localization of the patient's known meningioma, with comparable histological findings (H&E 20×).

Immunohistochemistry analysis of the hepatic lesion showed positive epithelial membrane antigen (EMA) and negative S100 protein (S100), consistent with the meningiomatous nature of metastasis. Progesterone receptor (PR), SSTR2A, STAT6, and cytokeratin panels were not performed which we acknowledge as a limitation in fully excluding other rare tumor types. Following this diagnosis, the patient was started on Sandostatin and Everolimus 5 mg daily and a left frontoparietal craniotomy was performed in September 2022, which revealed an atypical meningioma (Grade 2, 2021 WHO CNS classification) with areas of anaplastic transformation (Grade 3, 2021 WHO CNS classification) and a variable Ki‐67 proliferation index with hotspots of 15%–20% (Figure 5). Postoperatively, stereotactic radiotherapy (SRT) of 25 Gy in 5 fractions was administered to target multiple residual intracranial lesions.

### 2.4 Clinical Outcomes

From that time onward, follow‐up evaluations demonstrated a stable hepatic metastatic lesion. The most recent imaging showed an oval hypodense lesion at the hepatic dome measuring 4 cm, comparable with the previous studies, with no intra‐ or extrahepatic biliary dilatation and no new lesions. In contrast, intracranial disease continued to progress intermittently and was managed with repeated surgical resections or radiotherapy.

### 2.5 Follow‐Up

Clinically, the patient did not return to his baseline state. He continued to experience intermittent episodes of transient aphasia, mild confusion, and incomplete recovery of normal cognitive function. Despite multiple interventions, the meningiomatous lesions progressed, and the patient died in September 2023 during surgery for resection of a recurrent lesion.

The patient's clinical course from August 2010 to September 2023, including surgical interventions and pathological findings, is summarized in the table below (Table 1).

**TABLE 1:** Surgical interventions, WHO Grade, and Ki‐67 progression in the patient.

Surgical date	Pathological diagnosis	WHO grade	Ki‐67	Clinical presentation
August 2010	Transitional meningioma	1	NM	Initial diagnosis and resection
September 2017	Meningothelial meningioma	1	10% (Hotspots 25%–30%)	First recurrence, second resection
July 2019	NM	1	NM	Second recurrence, third resection
February 2020	Atypical transitional	2	10%	Fourth resection: progression to Grade II
September 2022	Anaplastic transformation	3	15%–20%	Fifth resection; focal Grade III histology
November 2022	Metastatic meningioma	2 (Met)	4%–7%	Hepatic metastasis detected via biopsy

## 3 Discussion

Meningiomas are the most common primary intracranial tumors and can spread through hematogenous, lymphatic, or cerebrospinal fluid routes. Deposition of tumor cells extracranially can be explained by either the passage of tumor cells from venous circulation into organs or through the (vertebral) meningorachidian venous system [6, 7]. Multiple risk factors have been described associated with an increased risk of distant metastasis. For instance, pathologic criteria associated with higher grades, like the histologic grade of the tumor, mitotic rate, nuclear atypia, high cellularity, presence of small cells, prominent nucleoli, anaplastic features, and presence of foci of necrosis, are thought to favor tumor dissemination [4]. Clinical variables associated with this event were male gender and large tumor size [5]. Additionally, other factors such as tumor localization, vicinity to venous drainage, and repeated surgical excisions were attributed to an increased risk of extracranial metastasis [8].

Despite the abovementioned risk factors for meningioma spread, extracranial meningioma metastasis remains extremely rare occurring at a rate of less than 1%, and especially to organs such as the liver [2].

### 3.1 Review of Reported Extracranial Metastatic Meningiomas

To contextualize our case, we reviewed the literature from the past decade (2016–2024) on extracranial metastases of meningioma. We excluded reports describing scalp involvement, as this could represent local extension rather than true metastasis. Only cases of intracranial meningiomas were included, excluding orbital or spinal origins and those in which metastases were the initial presenting feature. Table 2 presents the data reviewed from the past decade (Table 2).

**TABLE 2:** Summary of reported cases of extracranial meningioma metastases over the past decade. Abbreviations: M: Male; NM: Not mentioned; ICP: Intracranial pressure.

Author/Year	Patient age/sex	Primary tumor location	WHO grade	Location of metastasis	Time to metastasis	Clinical presentation	Treatment for metastasis	Outcome/follow‐up
Our case, 2025	73/M	Left frontal	II	Liver	12 years	Fever Altered LFTs	Sandostatin, everolimus	Death
Sheng et al., 2020 [9]	55/M	Right parietal	II	Liver	6 years	Right upper abdomen pain	NM	NM
S J Y Chew et al., 2021 [10]	68/F	NM	II	Lymph nodes	Sixteen years	Right facial mass during orbital exenteration and neck dissection	Surgery	Death
Ghomari et al., 2023 [11]	66/F	falcine meningioma	II	Liver and iliac bone	9 years	Asymptomatic, incidental imaging	NM	NM
Utsumi et al., 2022 [12]	75/M	Left Convexity	II	Lung	2 years	Asymptomatic, incidental imaging	Video‐assisted thoracoscopic left lower lobectomy.	Alive, stable
Yuki mitani et al., 2024 [13]	55/M	NM	II	Femur	5 years	Pain, limited mobility	Surgical excision + radiotherapy	Stable, no recurrence
Inouss et al., 2025 [14]	60/F	Cerebellar	III	Adrenal gland	At diagnosis	Fatigue, incidental imaging	Bevacizumab	Alive, monitored
Matsumura et al., 2021 [15]	75/M	NM	II	Sternum	29 years	Swelling of the anterior chest	Resection	Recurrence in the thoracic cavity and pericardium Metastasis to the abdominal cavity, death
Ward et al., 2018 [16]	48/M	Frontal bilateral	II	C7 vertebra	6 years	Pain in the neck and left upper extremity	Resection and Gamma knife therapy	Resolution of the neurologic deficits and stabilization
Ningning He et al., 2020 [17]	38/M	Parietal bilateral	III	Lung and pleura	7 years	Incidental imaging	Radiation, Chemotherapy (50 Gy + 4 cycles Taxol), Gamma knife radiosurgery	Lung recurrence, palliative treatment
Alper Dincer et al., 2020 [18]	41/M	Midline	I	Lung	At diagnosis	Incidental imaging	Follow‐up	Stable
Destiny D Bailey et al., 2023 [19]	69/M	Middle cranial fossa	II	Axial and appendicular skeleton, liver, kidney, lung	8 years	Weight loss, decreased appetite, abdominal fullness, and epigastric pain	Chemotherapy (everolimus and octreotide), Luthatera	Death
Hanna et al., 2020 [20]	67/M	Left Frontoparietal	I	Vertebral bodies	10 years	Bone (L3/T11) Back pain	Radiation	NM
Borni et al., 2024 [21]	31/M	Right extra‐axial parieto‐temporo‐occipital lesion	II	Liver	3 years	Raised ICP; Liver masses	Radiation, bevacizumab	Favorable

A total of 13 cases were identified: 10 males and 3 females. Most were grade 2 or anaplastic meningiomas, with two rare grade 1 cases. The interval between initial diagnosis and the detection of metastases ranged from a few months to over a decade, in our case, the patient had the liver metastatsis 12 years after the initial diagnosis of meningioma and after five consecutive surgical excisions. Recent reports a trend toward earlier detection of extracranial metastases using positron emission tomography/computed tomography (PET/CT) [22]. Five cases were identified incidentally through imaging. Surgical resection remained the treatment of choice for oligometastatic disease, which represented the most common extracranial presentation; only two patients had multiple metastatic sites.

Emerging therapies, including systemic treatments and radiotherapy, have shown a role in disease control. Among the reported cases, three patients died (two from recurrence of the primary tumor and one from disease progression), while outcomes were not reported in two cases. These findings emphasize the importance of active surveillance and tailored management for patients at risk of extracranial spread.

Our patient developed extracranial metastases in November 2022, around the same time as intracranial recurrence of his grade 2 meningioma, which showed anaplastic transformation and a Ki‐67 increase from 10% to 15%–20%.

In general, a Ki‐67 of 4% indicates a benign tumor and a Ki‐67 of 10% to 15% suggests aggressive behavior [23]. Upon re‐evaluation, the 2017 tumor specimen was reviewed according to the 2021 WHO CNS classification and confirmed as Grade I, with no evidence of brain invasion. Molecular testing, such as TERT promoter mutation or CDKN2A/B deletion analysis, was not performed on earlier specimens. The apparent changes from transitional to meningothelial histology and back likely reflect interobserver variability and intratumoral heterogeneity rather than true histological transformation, as a single biopsy or even a subtotal resection may fail to capture the most aggressive portion of the tumor. Interestingly, the liver metastasis biopsy demonstrated a lower Ki‐67 index. Consequently, while the liver metastasis was detected before overt anaplastic transformation, this observation should be interpreted cautiously considering a hypothesis that the possibility of metastatic spread may occur before or alongside histological progression and warranting further investigation. Detection of metastases should prompt brain MRI to assess tumor progression and guide management.

### 3.2 Risk‐Based Screening for Extracranial Metastases in Atypical Recurrent Meningioma

The fifth edition of the WHO Classification of Tumors of the CNS (2021) introduced the new tumor types and subtypes, incorporating molecular and epigenetic profiles. Grade 2, or atypical, meningiomas are defined by a mitotic index of 4–19 mitoses per 10 high‐power fields, evidence of brain invasion, or the presence of at least three of five histopathological features: spontaneous or geographic necrosis, prominent nucleoli, increased cellularity, small cells with a high nucleus‐to‐cytoplasm ratio, and a patternless (sheet‐like) growth pattern. Among grade 2 (atypical) meningiomas, the 5‐year disease‐free survival is approximately 50%, reflecting their heterogeneous biological behavior. Histopathological features such as brain invasion, sheeting, and a high mitotic index have been associated with an increased risk of recurrence [24]. Repeated surgical interventions may also mechanically facilitate the entry of tumor cells into the jugular and paravertebral venous systems, potentially contributing to systemic dissemination [25]. In our patient, the high mitotic rate may indicate an increased metastatic potential, and repeated surgical procedures may have further contributed to tumor spread. Recent studies have proposed that systemic imaging should be considered in patients with multiple recurrences or symptoms suggestive of metastasis to identify extracranial disease [2]. Currently, there are no formal guidelines for systemic screening in meningioma patients, yet studies have shown that inpatients with three or more recurrences, the incidence of metastasis can reach nearly 2% for Grade 2 and over 8% for Grade 3 tumors [2]. Although gallium‐68 DOTA‐Tyr3‐octreotide (Ga‐DOTATOC) PET/CT is not yet considered a standard imaging modality for whole‐body staging in patients with high‐grade meningiomas, it offers important advantages for treatment planning, response assessment, and theranostic stratification, and its clinical relevance is increasingly recognized [26]. Although hepatic metastasis remains rare, the increasing use of DOTATATE PET/CT may improve detection rates, potentially altering the prognosis for high‐risk patients. DOTATOC PET/CT staging at diagnosis or recurrence can guide treatment decisions in malignant meningiomas and could have been beneficial for the surveillance of our patient [27]. Future classifications and management guidelines may benefit from integrating these histopathologic risk factors and imaging to better identify grade 2 meningiomas at higher risk for systemic spread.

### 3.3 Current and Emerging Treatments for Metastatic Meningioma

Treatment of metastatic meningioma can be very challenging due to the rarity of cases and lack of consensus. For inoperable or recurrent meningiomas, the European Association of Neuro‐Oncology (EANO) guidelines recommend individualized treatment strategies. Combined treatment strategies incorporating surgery with radiosurgery or fractionated radiotherapy are increasingly used, although the optimal timing, modality, and radiation dose remain subjects of ongoing debate [28].

When local therapies are no longer feasible, systemic pharmacotherapy becomes an important consideration, particularly in the setting of metastatic or progressive disease. Radionuclide therapy targeting somatostatin receptors, with or without everolimus, has shown promising results in aggressive meningiomas, as demonstrated in the CEVOREM trial [29]. In our patient, this approach resulted in stabilization of the hepatic lesions, whereas the intracranial meningioma continued to progress. This differential response can be explained by the pharmacokinetic properties of both Sandostatin (octreotide) and everolimus. Everolimus, an mTOR inhibitor, has limited penetration across the blood–brain barrier and therefore achieves more effective drug exposure outside the central nervous system. The reduced intracranial response may also reflect the predominantly cytostatic rather than cytotoxic effects of both agents, as well as heterogeneous or insufficient somatostatin receptor subtype 2 (SSTR2) expression within the tumor. Although Sandostatin can cross the blood–brain barrier, its efficacy depends on SSTR2 expression, which may vary between intracranial and extracranial sites. Differences in receptor expression and drug distribution likely contribute to heterogeneous therapeutic responses, with potentially greater efficacy outside the CNS. The combination of both agents may partially overcome these limitations through additive and complementary mechanisms of action [30]. Furthermore, bevacizumab has shown promise in controlling aggressive meningiomas [31]. By inhibiting angiogenesis, a key process driving tumor growth, progression, and systemic spread, it may also help reduce metastatic potential and aid in managing extracranial disease. Finally, this case highlights the cumulative risks associated with repeated surgical resections. The patient ultimately died from massive intraoperative hemorrhage during the sixth brain surgery, underscoring how repeated operations can lead to scar formation and distorted vascular anatomy, paradoxically turning attempts to control the local disease into the cause of mortality.

## 4 Conclusion

Metastatic meningiomas represent a significant yet likely underestimated clinical challenge. As with our patient, similar cases show poor outcomes, although death rarely results from extracranial metastases. To optimize patient management, timely identification of individuals at risk for metastasis is crucial. Further research is needed to elucidate the underlying mechanisms, particularly considering the updated WHO classification, to determine whether specific molecular alterations contribute to metastatic potential. This understanding could guide the development of targeted screening strategies, as routine screening for all meningioma patients is not feasible. Prognosis is primarily influenced by the difficulty in achieving durable control of the primary tumor, yet management of metastatic sites is equally important to prevent further deterioration of outcomes. Ultimately, a consensus is needed on optimal approaches for screening, risk stratification, and management of patients with metastatic meningioma.

## Author Contributions

**Tala Najdi:** conceptualization, data curation, formal analysis, investigation, project administration, validation, writing – original draft. **Sami Abi Farraj:** data curation, formal analysis, writing – original draft. **Pierre El Sett:** formal analysis. **Clarisse Kattan:** investigation, methodology, project administration. **Antoine El Sett:** conceptualization, data curation. **Antoine Chartouni:** project administration, writing – original draft. **Patrick Ghorayeb:** conceptualization, resources, software. **Ghadi Dagher:** conceptualization, data curation. **Rhea Khazen:** data curation, resources. **Fares Azoury:** supervision, validation. **Joseph Kattan:** supervision, validation, visualization, writing – review and editing. **Hampig Kourie:** project administration, visualization.

## Funding

The authors have nothing to report.

## Ethics Statement

The authors have nothing to report.

## Consent

Written informed consent was obtained from the patient for publication of this case report and any accompanying images.

## Conflicts of Interest

The authors declare no conflicts of interest.

## Data Availability

No datasets are publicly available. All information relevant to this case report is contained within the article.
